# Identifying cancer prognostic modules by module network analysis

**DOI:** 10.1186/s12859-019-2674-z

**Published:** 2019-02-18

**Authors:** Xiong-Hui Zhou, Xin-Yi Chu, Gang Xue, Jiang-Hui Xiong, Hong-Yu Zhang

**Affiliations:** 1 Hubei Key Laboratory of Agricultural Bioinformatics, College of Informatics, Huazhong Agricultural University, Wuhan, 430070 People’s Republic of China; 2 State Key Laboratory of Space Medicine Fundamentals and Application, China Astronaut Research and Training Center, Beijing, People’s Republic of China; 3 Lab of Epigenetics and Health Tracking Technology, Space Institute of Southern China, Shenzhen, People’s Republic of China

**Keywords:** Module network, Cancer prognosis, GeneRank, Drug targets

## Abstract

**Background:**

The identification of prognostic genes that can distinguish the prognostic risks of cancer patients remains a significant challenge. Previous works have proven that functional gene sets were more reliable for this task than the gene signature. However, few works have considered the cross-talk among functional gene sets, which may result in neglecting important prognostic gene sets for cancer.

**Results:**

Here, we proposed a new method that considers both the interactions among modules and the prognostic correlation of the modules to identify prognostic modules in cancers. First, dense sub-networks in the gene co-expression network of cancer patients were detected. Second, cross-talk between every two modules was identified by a permutation test, thus generating the module network. Third, the prognostic correlation of each module was evaluated by the resampling method. Then, the GeneRank algorithm, which takes the module network and the prognostic correlations of all the modules as input, was applied to prioritize the prognostic modules. Finally, the selected modules were validated by survival analysis in various data sets. Our method was applied in three kinds of cancers, and the results show that our method succeeded in identifying prognostic modules in all the three cancers. In addition, our method outperformed state-of-the-art methods. Furthermore, the selected modules were significantly enriched with known cancer-related genes and drug targets of cancer, which may indicate that the genes involved in the modules may be drug targets for therapy.

**Conclusions:**

We proposed a useful method to identify key modules in cancer prognosis and our prognostic genes may be good candidates for drug targets.

**Electronic supplementary material:**

The online version of this article (10.1186/s12859-019-2674-z) contains supplementary material, which is available to authorized users.

## Background

The identification of prognostic genes that can distinguish the prognostic risks of cancer patients is essential for the study of cancer. These genes could be used to predict the prognosis of cancer patients [1, 2]. Additionally, the prognostic genes may be essential in the biological process of cancer progression and metastasis and thus may be potential drug targets [3, 4]. However, most of the published signatures suffer poor generalization [5]. That is, the prognostic genes selected from one data set are not correlated with the prognostic risks in other data sets [6]. This phenomenon may be due to the high heterogeneity of cancer [7]. Therefore, the selected genes whose expression levels are correlated with the prognostic risks in one data set may be passengers rather than drivers in others.

Based on the hypothesis that genes involved in a certain functional gene set (i.e., GO term or Pathway) may be more stable, a few works attempted to identify prognostic gene sets based on GO term [8], Pathway [9] and modules in the PPI (protein-protein interaction) network [10–12] or a gene co-expression network [11]. In addition, the prognostic modules (gene sets) outperform the gene signatures [13]. Therefore, it seems that gene modules rather than gene signatures are more promising in cancer prognosis.

As we know, cross-talk among pathways is common [14], and understanding the cross-talk between pathways is essential for the study of more complex systems [15, 16]. However, most previous works have ignored the cross-talk among the modules, which may result in neglecting the driver modules in cancer prognosis.

In this work, based on fact that the dense clusters in co-expression networks may serve as a functional unit to influence the prognosis of cancer patients, we first constructed a gene co-expression network using the gene expression data of cancer patients. Then, the modules that were dense clusters in the network were detected. Adopting a similar strategy as in a previous work [16], we identified cross-talks between every two modules by testing whether the number of edges between the two modules are significant compared with the background distribution of the edges’ number of two random gene sets. Then, all cross-talks among the modules could constitute a module network. To identify the essential modules in cancer prognosis, we first calculated the prognostic correlation of each module by a resampling method. Then, the algorithm of GeneRank [17], which takes the module network and the prognostic correlations of all the modules as input, was applied to prioritize the prognostic modules. The prognostic modules were validated by survival analysis in various data sets. In addition, we also performed the enrichment analysis of these genes involved in modules with curated cancer genes and drug targets to validate the prognostic modules. The evolutionary information of cancer driver genes is helpful for the construction of cancer prognosis prediction models [18, 19]. Therefore, we also investigated the evolutionary feature of our genes involved in the prognostic modules. Furthermore, our method was applied in three kinds of cancers (ovarian cancer, breast cancer and lung adenocarcinoma) and was compared with the state-of-the-art methods.

## Methods

### Data sets and pre-processing

We applied our method to data sets of ovarian cancer, lung adenocarcinoma and breast cancer. All the data sets contain gene expression data and prognostic information (time of death and death status) of cancer patients. In this work, the data set of lung cancer from TCGA (The Cancer Genome Atlas) was measured by RNA-seq, and the gene expression data of all the other data sets was measured by genechip. For all gene expression data, the probes were mapped to Entrez Gene ID, and the expression levels of the probes for each gene were averaged.

In ovarian cancer, 1432 samples from two data sets were collected (the detailed information of the two data sets was shown in Additional file 1: Table S1). Among these data sets, 300 samples from TCGA were randomly selected for the training data set and the other 267 samples from TCGA were assigned to the test data set. The other data set was used as independent data set.

In lung adenocarcinoma, 535 samples from TCGA and 443 samples from GSE68465 were used in this work. Among these samples, 300 samples from TCGA were randomly selected for the training data set, and the other 235 samples from TCGA were assigned to the test data set. All the samples in GSE68465 [20] were set as the independent data set.

For breast cancer, a merged data set [21] containing 855 samples was used in this work. In this data set, GSE2034 [22] was set as the independent data set. Of the other 569 samples, 300 samples were randomly chosen for the training data set, and the others were assigned to the test data set.

We collected the cancer genes from COSMIC and Sanger [23]. The adaptation diseases and the target information of drugs were obtained from the TTD (Therapeutic Target Database) [24], the DGIdb (Drug-Gene Interaction Database) [25] and DrugBank [26, 27].

### Construction of the gene co-expression network using a rank-based method

The Pearson correlation coefficient was applied to calculate the correlations of the expression levels between every two genes. Based on the correlation coefficient, a rank-based method was used to construct the gene co-expression network [28]. As we know, genes in one functional pathway may be strongly mutually co-expressed, but genes in another functional pathway may be weakly co-expressed [28]. Therefore, it may be reasonable to construct the gene co-expression network based on the rank-based method rather than the value-based method. The former method selects the co-expression genes of each gene by the rank of the correlations, and the latter method identifies a gene’s neighbors based on a threshold of the correlations. In this work, adopting a similar strategy to the rank-based method [28], for each gene, we selected the 10 most correlated genes as its neighbors, and all the gene pairs constitute the gene co-expression network.

### Network visualization and module detection

We used Cytoscape 3.5.3 to visualize the co-expression networks and the module networks, and the MCODE [29] plug-in for Cytoscape was applied to detect the dense clusters in the network. In this work, only the modules containing no less than 5 nodes were retained.

### Construction of the module network using the permutation method

In the co-expression network, if the number of edges across two modules is significantly high, then there may be cross-talk between the two modules. The significance of the cross-talk between every two modules is calculated by a permutation test, which is shown as follows:

1. The number of the edges across the two modules in the gene expression network is calculated.
2. Two random gene sets, which contain the same number of genes as the two real modules, are selected from the gene co-expression network. Then, the number of edges across the random gene sets is calculated.
3. The procedure in step 2 is repeated 1000 times, and the edge numbers across the random gene sets are set as the null hypothesis distribution. Based on the null hypothesis distribution, the *p*-value of the cross-talk between the two modules is calculated.

Based on the permutation test, all significant module pairs with *p*-values less than 0.05 could constitute a module network.

### Calculation of the prognostic capability of the modules using a resampling method

The correlation between the gene expression levels of the modules and the prognostic risks of cancer patients could be calculated by cox regression. In order to obtain a more stable result for cox regression, a resampling method, which aims to generate more training data sets for cox regression, was proposed. For each module, the results for 400 cox regressions in the training data sets by resampling, were used to evaluate its prognostic capability, which would be used as an input in the module prioritization algorithm.

First, the expression levels of the genes in the modules were averaged as the statistical values of the corresponding modules. The statistical values for each module in the corresponding patient could be calculated by the follow equation.

1 $$ s=\frac{\sum \limits_{i=1}^n{e}_i}{n} $$

Here, *n* is the number of genes in the module. *e*_*i*_ is the expression level of the ith gene of the module in the corresponding patient. Therefore, the statistical value of this module in the corresponding patient could be calculated.

Then, 90% of the samples in the training data set were randomly selected. In the chosen data set, the Cox proportional hazards regression was applied to calculate the relationship between each module’s statistical value and the prognostic risks (time of death and death status) of the selected patients.

Finally, we repeated the procedure 400 times, and the significant frequency, that is, the number of times that the module’s cox *p*-value was less than 0.05 in the 400 runs, was set as the prognostic capability of the module. The significant frequency of each module could characterize the prognostic stability of it. Furthermore, the average Cox coefficient of each module in the 400 runs is set as the final Cox coefficient of the module.

### Prioritizing modules using the algorithm of GeneRank

The GeneRank algorithm [17] succeeded in identifying key genes from the biological network. Here, we applied it to prioritize the essential modules in prognosis from the module network. The algorithm is described as follows:

2 $$ {r}_j^n=\left(1-d\right){p}_j+d\sum \limits_{i=1}^N\frac{{\mathrm{w}}_{\mathrm{i}\mathrm{j}}{{\mathrm{r}}_{\mathrm{i}}}^{\mathrm{n}\hbox{-} 1}}{\deg {ree}_{\mathrm{i}}} $$

Here, ${r}_j^n$ is the importance (prognostic capability) of the module *j* after *n* iterations; *p*_*j*_ is the initial importance, which is calculated by the resampling method; *w*_*ij*_is equal to 0 or 1, with 1 indicating the existence of cross-talk between module *i* and module *j*, and 0 indicating no interaction between the two modules; deg*ree*_*i*_ is the number of neighbors of module *i* in the module network; *N* is the module number in the network; and *d* (*0 ≤ d < 1*) is a constant, where a larger *d* indicates that the importance of the modules is dependent more on the topological structure of the network, and a smaller *d* indicates that the importance of the modules depends more on the initial importance of the modules. Here, we adopted the same strategy as a previous work [30] and set *d* as 0.70.

As proved in this work [17], the above iteration corresponds to Jacobi on the system

3 $$ \left(I-{dW}^T{D}^{-1}\right)r=\left(1-d\right)p $$

Here, *I* is the identity matrix, *W* is the adjacency matrix of our module network; *D* =  *diag* (deg*ree*_*i*_ ), and *p* is a vector (*N × 1*) that contains the initial importance of the *N* modules in the network. By solving this equation, the vector *r* (*N × 1*), which contains the final importance of all the nodes in the network, could be obtained.

### Survival analysis using selected modules

Based on the prognostic modules, GGI [31] was applied to calculate the prognostic risk of each patient:

4 $$ Risk\kern0.17em Score=\sum {s}_i-\sum {s}_j $$

Here, *s*_*i*_ is the statistical value (the average value of all the genes’ expression levels in the module) of the module whose Cox coefficient is positive, and *s*_*j*_ is the statistical value of the module whose Cox coefficient is negative. Then the patients in the data set were divided into two groups with the same number of patients according to their prognostic risks. Finally, the log rank test was performed to test whether there is a significant difference in the real survival risks between the two groups.

Furthermore, a *discrimination score* (*Dscore*) was defined to evaluate the distinguishing capability of the prognostic modules across various data sets, which is described as follows:

5 $$ Dscore=-\sum \limits_{i=1}^n{\log}_{10}\left(p-{value}_i\right) $$

In this equation, *p* − *value*_*i*_ is the log rank *p*-value in the *i*th data set, and *n* is the number of cancer data sets.

### Enrichment analysis

The hypergeometric test was applied to test whether the intersection of the genes in the prognostic modules and the known cancer genes (or the drug targets) is significant, which was calculated as follows:

6 $$ p- value=F\left(x/M,K,N\right)=1-\sum \limits_{\mathrm{i}=0}^{x-1}\frac{\left(\begin{array}{l}\mathrm{K}\\ {}\mathrm{i}\end{array}\right)\left(\begin{array}{l}\mathrm{M}\hbox{-} \mathrm{K}\\ {}\mathrm{N}\hbox{-} \mathrm{i}\end{array}\right)}{\left(\begin{array}{l}\mathrm{M}\\ {}\mathrm{N}\end{array}\right)} $$

Here, *x* is the number of genes in the intersection set, *M* is the number of genes in the universal set, *K* is the number of genes in the modules and *N* is the number of cancer genes (drug targets).

## Results

### The module networks of the three cancers

The module network would reveal cross-talks among the modules. Therefore, the module network could facilitate the identification of key modules in cancer prognosis. In this work, we propose a new method to construct the module network. First, based on the gene expression data of cancer patients, a rank-based method was used to construct a gene co-expression network. Then, the dense clusters, which were communities in the network, were detected as modules. Next, a permutation test was proposed to identify cross-talks among the modules. In this work, we applied it in ovarian cancer, breast cancer and lung adenocarcinoma, respectively. The module networks of the three cancers are shown as follows.

#### The module network of ovarian cancer

Using the gene expression profiles of ovarian cancer patients in TCGA, a gene co-expression network was constructed. In this network, there are 15,406 nodes and 154,060 edges, and the average number of neighbors of the genes in the network was 16.67. The power-law fit of the nodes’ degrees with the number of nodes showed that the network was scale-free, with a correlation of 0.902 and an R-square of 0.925 (Additional file 1: Figure S1).

Based on the co-expression network, 258 modules were detected. The genes within each module were densely connected with each other and rarely connected with other genes outside the module. After the identification of cross-talks among these modules, the module network of ovarian cancer was constructed (Additional file 1: Figure S2). As a result, 957 edges were identified among the 258 modules, and the average number of neighbors of the modules was 7.419, which may indicate that cross-talk among the modules were common.

#### The module network of breast cancer

For breast cancer, the gene expression data of all the samples in the merged data set [21] (except for the samples in GSE2034 [22]) was used to construct a co-expression network. As a result, 170,920 co-expression pairs among 17,092 genes were obtained, and the average number of neighbors of the genes in the network was 16.92. The power-law fit of the nodes’ degrees with the number of nodes showed that the network was also scale-free, with a correlation of 0.937 and an R-square of 0.945 (Additional file 1: Figure S3). The module network constructed based on the co-expression network contained 150 modules, with 614 edges among the 150 modules (Additional file 1: Figure S4).

#### The module network of lung adenocarcinoma

The gene expression profiles of lung adenocarcinoma patients from TCGA were used to construct a gene co-expression network. In the co-expression network, there were 12,153 nodes and 121,530 pairs. For the nodes in the network, the average number of co-expressed genes was 16.76. Similar to the co-expression networks of ovarian cancer and breast cancer, the co-expression network of lung adenocarcinoma was also scale-free, with a correlation of 0.899 and an R-square of 0.950 in power-law fit (Additional file 1: Figure S5). Based on the co-expression network, the module network of lung adenocarcinoma was also constructed. There were 181 modules and 593 edges in the network (Additional file 1: Figure S6). Furthermore, the average number of neighbors of each module was 6.701.

From these results, we can see that the module networks in all three cancers are dense, which may indicate that cross-talks among the modules are common.

### The prognostic modules of the three cancers

For the modules in each of the three cancers, the modules’ prognostic capabilities were calculated by the resampling method using the training data set of the corresponding cancer. Then, based on the module network and the modules’ prognostic capabilities, the algorithm GeneRank was applied to prioritize the modules in cancer prognosis for the three cancers, respectively. In each kind of cancer, top 5% of all the modules in the corresponding module network were selected as key modules. As a result, 13 modules in ovarian cancer (Additional file 1: Table S2), 8 modules in breast cancer (Additional file 1: Table S3) and 9 modules in lung adenocarcinoma (Additional file 1: Table S4) were identified, respectively.

### Survival analysis using the prognostic modules

To validate the prognostic modules, the survival analysis of the cancer data sets was performed for the three kinds of cancer. The results of the survival analyses of the three cancers are shown as follows.

#### Survival analysis in ovarian cancer

As described above, 13 modules in ovarian cancer were selected as key modules in the prognosis of ovarian cancer. Based on the gene expression data of the 13 modules’ statistical values, the prognostic risks of cancer patients could be calculated (Method). Then, a survival analysis could be used to test whether the patients in the low-risk group, calculated by our method, had longer survival times than the high-risk group.

**Fig. 1:**
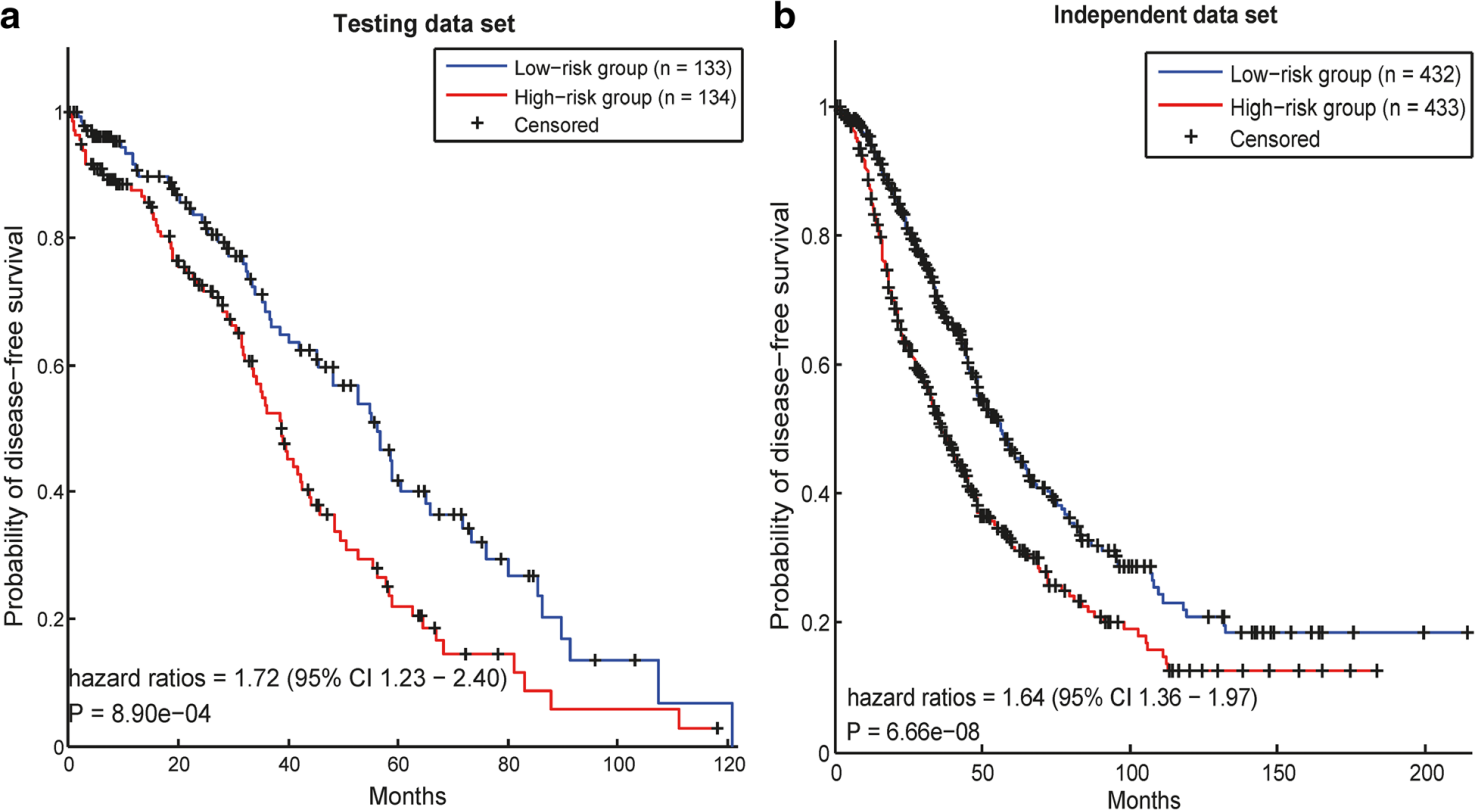
Survival analysis in ovarian cancer data sets. In each data set, the patients are divided into two groups according to their risk scores calculated by using the prognostic modules

In the testint data set (267 patients in TCGA), the HR (hazard ratio) of the two groups divided by our method was 1.72, and the log rank *p*-value was 8.90e-04 (Fig. 1a). In a previous work [32], a merged data set containing data for 1287 patients was collected to validate the prognostic signature in ovarian cancer. Here, after removing the redundant samples which were from the TCGA, we used the other 865 samples as independent data set. After that, we used our prognostic modules to predict the prognostic risks of all the patients in the independent data set. As a result, the HR of the two groups predicted by our method was 1.64, and the *p*-value was 6.66e-08 (Fig. 1b). These results indicate that our prognostic modules could discriminate the prognostic risks of cancer patients in ovarian cancer.

**Fig. 2:**
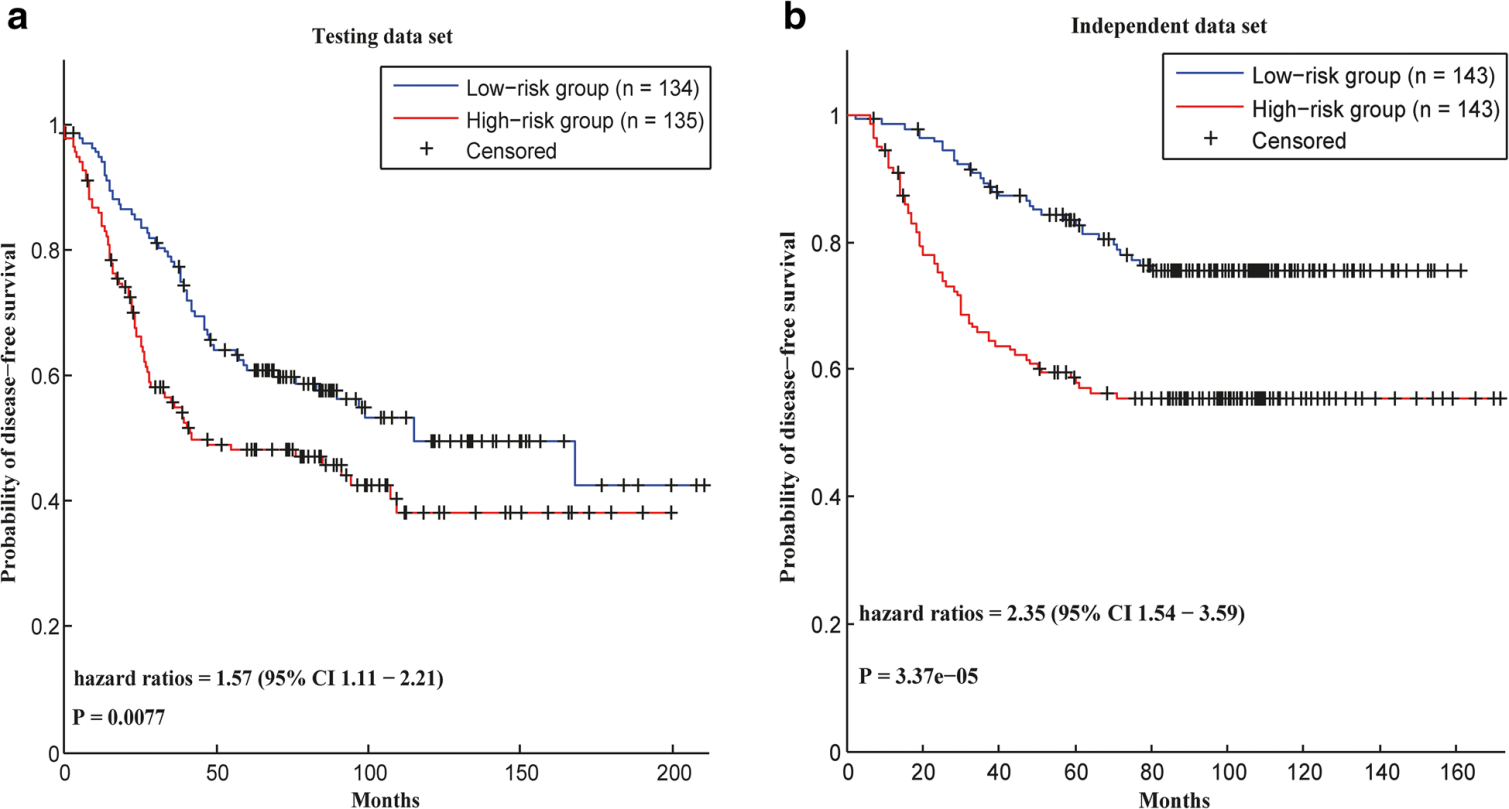
Survival analysis in breast cancer data sets. In each data set, the patients are divided into two groups according to their risk scores calculated by using the prognostic modules

#### Survival analysis in breast cancer

In breast cancer, a merged data set [21] containing 855 samples was used in this work. In this data set, all 286 samples from GSE2034 were set as the independent data set [22]. As to the other 569 samples, 300 patients were selected for the training data set, and the others were assigned to the test data set. In the training data set, 8 modules were selected as prognostic modules.

Then, the selected modules were used to calculate the prognostic risks of the cancer patients in the test data set and in the independent data set. In the test data set, the low-risk group had a significantly longer survival time, with an HR of 1.57 and a *p*-value of 0.0077 (Fig. 2a). Furthermore, the survival analysis in the independent data set also proved that our modules could distinguish the prognostic risks of cancer patients, with an HR of 2.35 and a p-value of 3.37e-05 (Fig. 2b), respectively.

Therefore, a conclusion can be drawn that the prognostic modules could distinguish the prognostic risks of cancer patients in breast cancer.

#### Survival analysis in lung adenocarcinoma

In lung adenocarcinoma, 300 samples from TCGA were selected for the training data set, and the other 235 patients were assigned to the test data set. In addition, all 443 samples in GSE68465 [20] were set as the independent data set. Using our method, 9 modules in the training data set were identified.

**Fig. 3:**
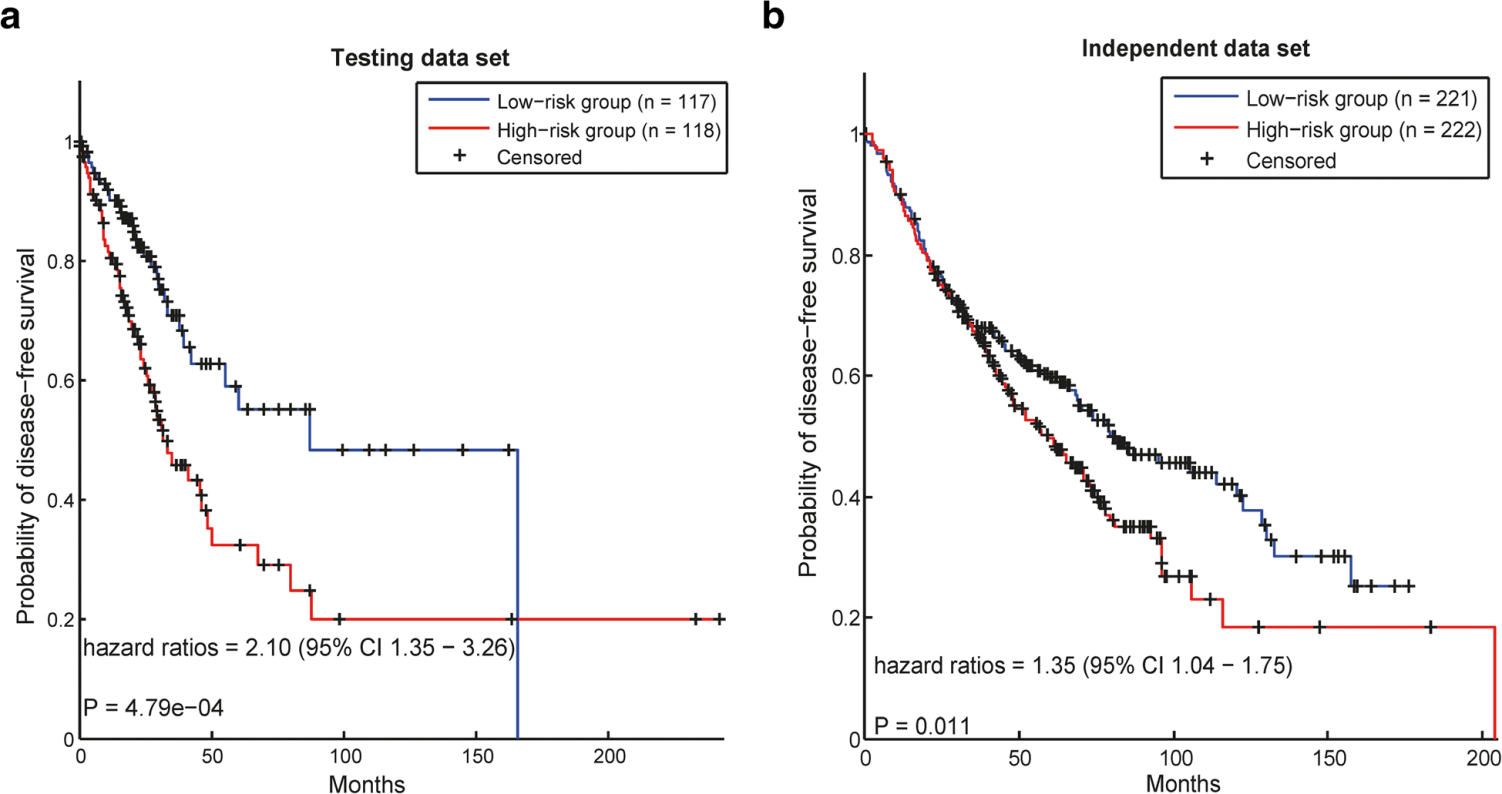
Survival analysis in data sets of lung adenocarcinoma. In each data set, the patients are divided into two groups according to their risk scores calculated by using the prognostic modules

Based on these modules, the prognostic risks of cancer patients in the test data set and the independent data set were calculated. The survival analysis in both data sets showed that our modules could distinguish the prognostic risks of cancer patients significantly, with HR values of 2.10 (*p*-value = 4.79e-04) in the test data set (Fig. 3a) and 1.35 (*p*-value = 0.011) in the independent data set (Fig. 3b).

From these results, we can see that our method could identify the prognostic modules in all three kinds of cancer. Additionally, these modules could distinguish the prognostic risks of cancer patients in a large number of patients from various data sets. As we know, the main problem of the traditional methods is that they cannot perform well in independent data sets. The good performance of our method has validated the superiority of our method.

### Comparison with other methods

As described above, the main hypothesis of our method is that the cross-talk among modules may influence the outcomes of cancer patients. Therefore, the module network may facilitate the identification of key modules in prognosis. To validate our method, the same numbers of modules as our prognostic modules, which were ranked by the resampling method, were selected as control modules. In a previous work [33], it has been proved that the random gene set may be also prognosis in multiple cancer types. Therefore, a permutation test was applied to test whether the performance of our method was better than the random gene sets, which contained the same number of genes as our modules. At last, we also compared the performance of our prognostic modules with the published signatures.

#### Comparing results in ovarian cancer

In ovarian cancer, 13 modules with the most correlated gene expression levels with the prognostic risks of cancer patients were identified by a resampling method. Then, the control modules were used to calculate the prognostic risks of cancer patients in the test data set and in the independent data set. The survival analysis showed that the control modules could distinguish the prognostic risks of cancer patients in both of the data sets (Additional file 1: Table S5). However, the control modules performed worse in both of the data sets compared with our prognostic modules. To evaluate the discrimination capability of the modules in various data sets, a Dscore was defined to characterize it (Method). As a result, the Dscore of our prognostic modules was 10.23, and the control modules achieved a Dscore of 7.78 (Table 1).

**Table 1:** Dscores of our prognostic modules, the control modules and the published signatures

	Ovarian cancer	Breast cancer	Lung adenocarcinoma
Prognostic modules	10.23	6.58	5.29
Control modules	7.78	5.05	4.76
Published signatures	3.55	4.50	0.87
42-gene signature	2.05	2.57	3.19

The *Dscore* is defined to characterize the distinguishing capability of the prognostic modules across various data sets. A higher *Dscore* means a better performance in prognosis

In addition, a permutation test was applied to validate our method. First of all, we randomly selected the same number of genes with that of our prognostic modules in ovarian cancer. After that, the random gene set was applied to calculate the prognosis risks of the patients in the same data sets, and a Dscore was calculated. At last, the process was repeated 1000 times, and a *p*-value was obtained by comparing the Dscore of our modules with the 1000 Dscores of the random gene sets. As a result, the p-value of our method is 0.10, which may indicate that our method is better than most of the random gene sets. In the meanwhile, the random gene set may be used to predict the prognosis of cancer patients with a much higher probability than expected [19]. This result may prove that the cross-talk among the modules could facilitate the identification of prognostic genes.

Furthermore, a 37-gene signature, which was identified in the literature [32], was also applied to predict the prognostic risks of cancer patients in these data sets. The signature could distinguish the prognostic risks in these data sets, with the log-rank *p*-values of 0.0076 and 0.037 in test data set and independent data set respectively (Additional file 1: Table S6). However, the Dscore showed that the signature performed worse compared with our prognostic modules and the control modules (Table 1).

In a previous work [6], 42 genes, which could predict the prognostic risks of cancer patients in multiple cancer types, were selected as prognostic markers. Here, we also compared the performance of our method with the 42-gene signature. This method performed well in the test data set (Additional file 1: Table S7). However, it couldn’t discriminate the prognostic risks in the independent data set. In addition, the Dsocre (2.05) of the 42-gene signature also showed our method performed better (Table 1).

#### Comparing results in breast cancer

For breast cancer, based on the resampling method, 8 modules were selected as control modules. Using the control modules, the patients in the test data set and the independent data set were predicted as low-risk or high-risk. The control modules could distinguish the prognostic risks in both data sets (Additional file 1: Table S8). However, our prognostic modules outperformed the control modules in both data sets. The Dscores of our prognostic modules and the control modules were 6.58 and 5.05, respectively.

We also compared the performance of our modules with that of the random gene sets by permutation test. As a result, the *p*-value was 0.0030. That is, out of 1000 random gene sets, only three were better than ours, which may indcate our method was significantly better than the random gene sets.

The 70-gene signature [34] is the most well-known gene signature in breast cancer. Here, we calculated the prognostic risks of cancer patients in the same data sets. The 70-gene signature performed well in both data sets (Additional file 1: Table S9), but the performance of our method was the better (Table 1). In addition, the 42-gene signature [6] was also used to do survival analysis in the breast cancer data sets. As a result, it couldn’t distinguish the risks of cancer patients in these data sets (Additional file 1: Table S10), and its Dscore was 2.57.

#### Comparing results in lung adenocarcinoma

In lung adenocarcinoma, 9 modules were identified by the resampling method. Using the 9 modules as control modules, patients in the test data set (TCGA) and the independent data set (GSE68465) were predicted as high-risk or low-risk. The control modules performed well in the test data set but poorly in the independent data set (Additional file 1: Table S11). In the independent data set, the log rank p-value of the prognostic risks between the two groups was 0.11.

In lung adenocarcinoma, 1000 random gene sets were also selected to perform survival analysis in the data sets of lung adenocarcinoma. As a result, p-value of the permutation test was 0.0080, which may validate our method.

In a previous work [35], 16 genes were used as markers to predict the prognostic risks of cancer patients in lung adenocarcinoma. In this work, we used this signature to calculate the prognostic risks in the same data sets of our modules. The gene signature could not discriminate the prognostic risks in both data sets (Additional file 1: Table S12). As to the performance of the 42-gene signature [6], it performed well in the testing data set. However, it couldn’t distinguish the prognositc risks of cancer patients in the independent data set (Additional file 1: Table S13) and its Dscore was 3.19 (Table 1). The Dscores of our prognostic modules, the control modules and the signatures showed that the performance of our modules was the best and that the gene signature was the worst.

From these results, in all three types of cancer, our prognostic modules, which were based on both the module network and the resampling method, outperform the control modules, random gene sets and the published signatures. The performance of the control modules was better than the gene signature. The strong performance of our prognostic modules not only revealed the superiority of our method but also validated the hypothesis that cross-talks among modules may influence the outcomes of cancer patients.

### Enrichment analysis with the curated genes

To validate the clinical value of the genes involved in our modules, we investigated the overlaps between our prognostic genes and the known cancer genes. Furthermore, the overlap between our prognostic genes and the targets of drugs for the corresponding cancer was also investigated. In addition, the genes involved in the control modules were assessed using the same analysis to evaluate our method.

**Fig. 4:**
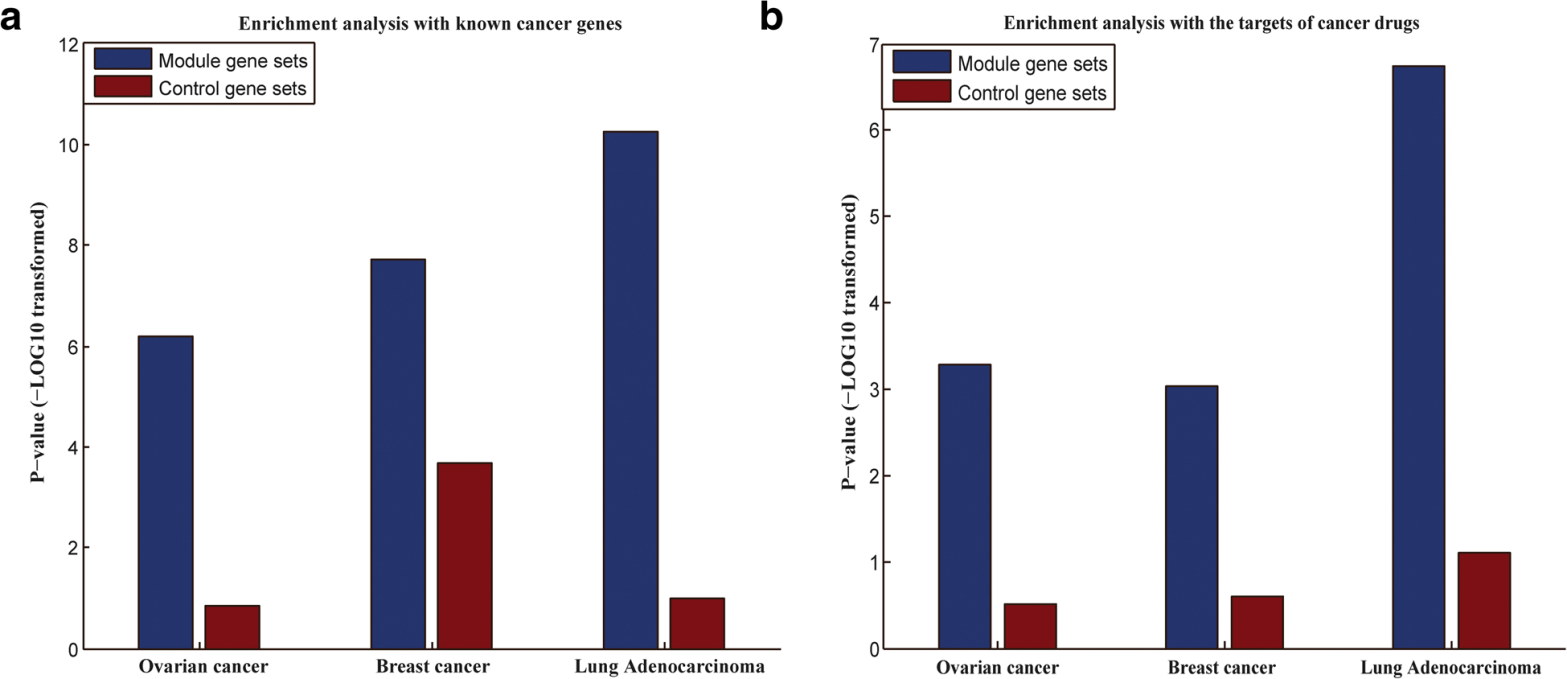
Enrichment analysis of the genes with the curated genes. **a** Enrichment analysis with known cancer genes. **b** Enrichment analysis with the targets of cancer drugs. The *p*-value was calculated by the hypergeometric test

The significance of the overlaps was calculated by the hypergeometric test. From Fig. 4, we can see that the genes involved in the prognostic modules are significantly enriched by known cancer genes and the targets of drugs for the corresponding cancer. In addition, our prognostic genes outperformed the control genes in all investigations.

The significance of the overlap between our prognostic genes and the known cancer genes may explain the distinguishing capability of our prognostic modules in cancer prognosis. An enrichment analysis of our prognostic genes with the targets of drugs for the corresponding cancer could prove the therapeutic value of our prognostic genes.

### The evolutionary origins of the genes in the selected modules

Cancer driver genes were observed to be enriched in genes originating from ancestors of multicellular organisms [36] and genes originating from Eukaryota [19]. Previous studies have shown that the cancer prognosis prediction models based on gene signatures, which are consistent with the evolutionary feature, are more accurate [18] and robust [19]. Therefore, it is of great interest to investigate that whether the origin of our prognostic genes is consistent with the evolutionary feature. The human gene age information was obtained from a previous work [37], which divided the genes into eight classes according to their origins. The origins of the genes include the first cellular organism, the common ancestor of Eukaryota and Archaea (Euk_Archaea), Eukaryota, Opisthokonta, Eumetazoa, Vertebrata, Mammals, and horizontally transferring from Bacteria (Euk + Bac).

**Fig. 5:**
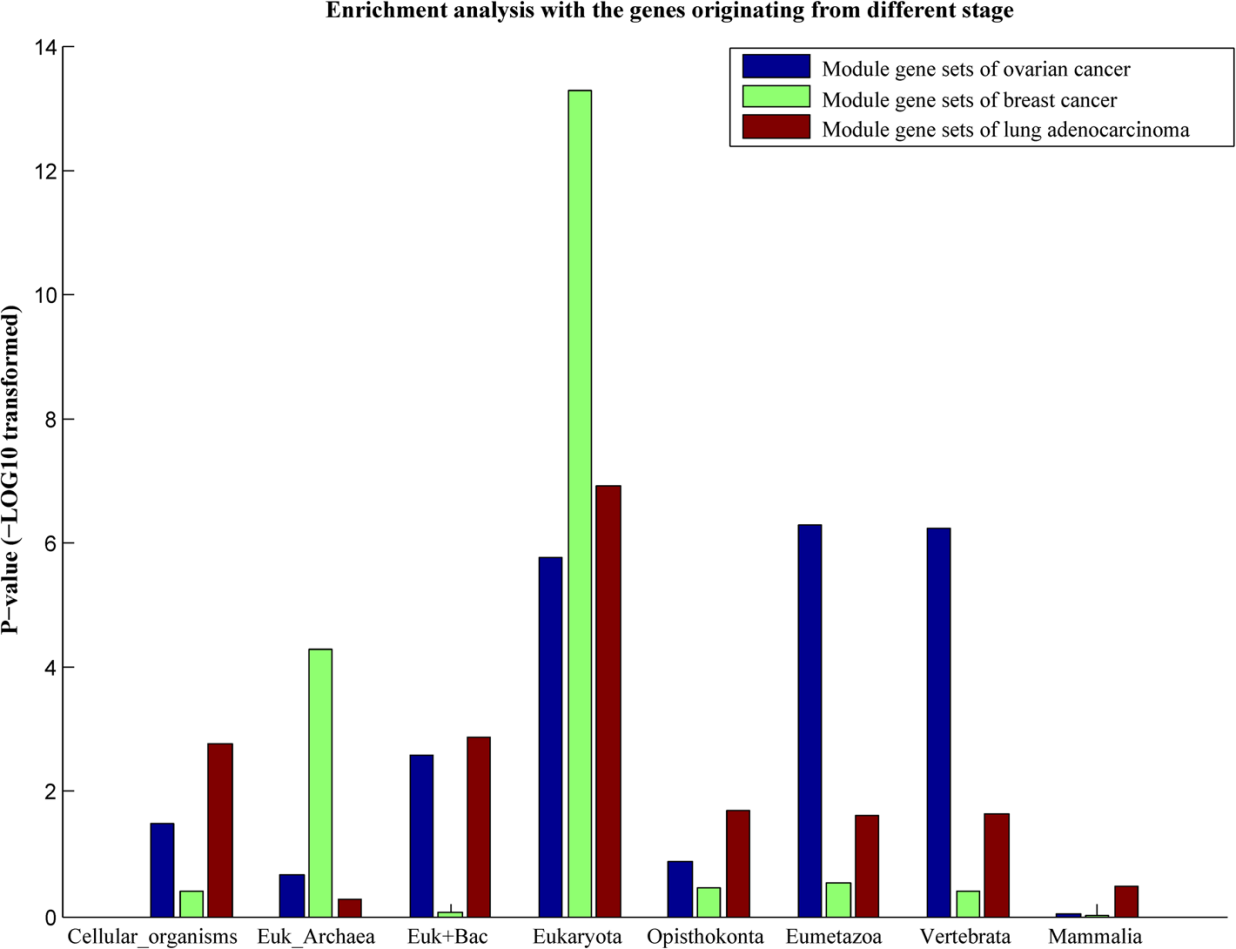
Enrichment analysis of our prognostic genes with the genes originating from different stages. The p-value was calculated by the hypergeometric test

For each cancer, the overlaps of the genes involved in the prognostic modules and the genes of different stages were calculated. The significances of the overlaps were calculated by a hypergeometric test (Fig. 5). From this result, we can see that the genes originating from the eukaryote were significantly enriched with the prognostic genes of all the three kinds of cancers. Our previous work also proved that the cancer driver genes were enriched by genes originating from Eukaryota [19]. In addition, in a latest work [38], the authors investigated the difference of expression levels (tumors vs. normal samples) of the genes originating from different stages and found that the genes from the stage of eukaryota are the most up-regulated ones, which is consistent with our results.

## Discussion

The identification of prognostic genes that can distinguish the prognostic risks of cancer patients remains a big challenge. Based on the hypothesis that functional gene sets may be more stable than the gene signature and that the investigation of the cross-talks among the functional gene sets may facilitate the prioritization of key modules (functional gene sets) in the prognosis of cancer patients, we propose a new method that involves both the interactions among modules (gene sets) and the prognostic capability of the modules to identify the prognostic modules in cancers.

We applied our method in three types of cancer, and the selected modules could distinguish the prognostic risks of cancer patients in a large number of data sets, including ovarian cancer, breast cancer and lung adenocarcinoma. The results showed that our prognostic modules performed better than the control modules, which were selected without using the module network. In addition, our prognostic modules also outperformed the published gene signature. All these results validate the hypothesis that the functional gene sets may be more stable than the gene signature and that the investigation of the cross-talks among the functional gene sets may facilitate the prioritization of key modules.

Furthermore, the biological meaning and the therapeutic value of the prognostic modules were also investigated. In all three cancers, the genes in the prognostic modules were significantly enriched with known cancer genes and the targets of drugs for the corresponding cancers, indicating that our prognostic genes may be good candidates as drug targets in cancer.

It is of great interest to investigate the evolutionary feature of the cancer driver genes. In this work, we also investigated the enrichment pattern of our prognostic genes with the genes originating from different stages of the evolutionary process. As a result, our prognostic genes were significantly enriched by the genes originating from the eukaryote in all the three types of cancer, which is consistent with the previous work [19].

The good performance of our method may be due to three reasons. First, our method is based on a reasonable hypothesis. Second, our method is data driven. Unlike the traditional method, the modules are always known functional gene sets (i.e., GO term or Pathway) and the modules in our method are dense clusters in the gene co-expression network. Therefore, our method may identify new modules. Third, our method applies suitable calculation models. For example, the algorithm of GeneRank takes advantage of both the topological structure of the module network and the statistical relationship between the modules’ gene expression data and the prognostic risks of cancer patients. As we know, the modules in the co-expression network are co-expressed with each other. Therefore, the use of the average value of the genes’ expression levels in the module as the statistical value of the module may remove the noise in the gene expression data.

## Conclusion

In conclusion, we proposed a useful method to identify key modules in prognosis. Our method could also be applied in the study of other biological problems as long as there are enough samples with transcriptome data.

## Additional file

Additional file 1: The file includes six figures (Figures S1–S6) and thirteen tables (Tables S1-S13). (DOCX 2399 kb)

## Abbreviations

HR: Hazard Ratio
TCGA: The Cancer Genome Atlas
