# Supplementary material for: Identifying cancer prognostic modules by module network analysis
Source: BMC Bioinformatics. 2019 Feb 18;20:85. doi: 10.1186/s12859-019-2674-z (PMC6380061; doi:10.1186/s12859-019-2674-z)
Supplement: Supplementary file 1 — The file includes six figures (Figures S1–S6) and thirteen tables (Tables S1-S13). (DOCX 2399 kb) [file 12859_2019_2674_MOESM1_ESM.docx]

**Identifying cancer prognostic modules by module network analysis**

Xiong-Hui Zhou^1^, Xin-Yi Chu^1^, Gang Xue^1^, Jiang-Hui Xiong^2,3^, Hong-Yu Zhang^1,*^

^1^ Hubei Key Laboratory of Agricultural Bioinformatics, College of Informatics, Huazhong Agricultural University, Wuhan 430070, P. R. China.

^2^ State Key Laboratory of Space Medicine Fundamentals and Application, China Astronaut Research and Training Center, Beijing, P. R. China.

^3^ Lab of Epigenetics and Health Tracking Technology, Space Institute of Southern China, Shenzhen, P. R. China.

Email addresses:

X.H.Z.: zhouxionghui@mail.hzau.edu.cn

X.Y.C.: chuxy@webmail.hzau.edu.cn

G.X.: [18202748974@163.com](mailto:18202748974@163.com)

J.H.X.: xiongjh77@163.com

H.Y.Z.: zhy630@mail.hzau.edu.cn

*To whom correspondence should be addressed.

The file includes six figures (Figure S1 – Figure S6) and thirteen tables (Table S1- S13). All the data and code for this work is available at <http://ibi.hzau.edu.cn/MNA/>.

## Figures


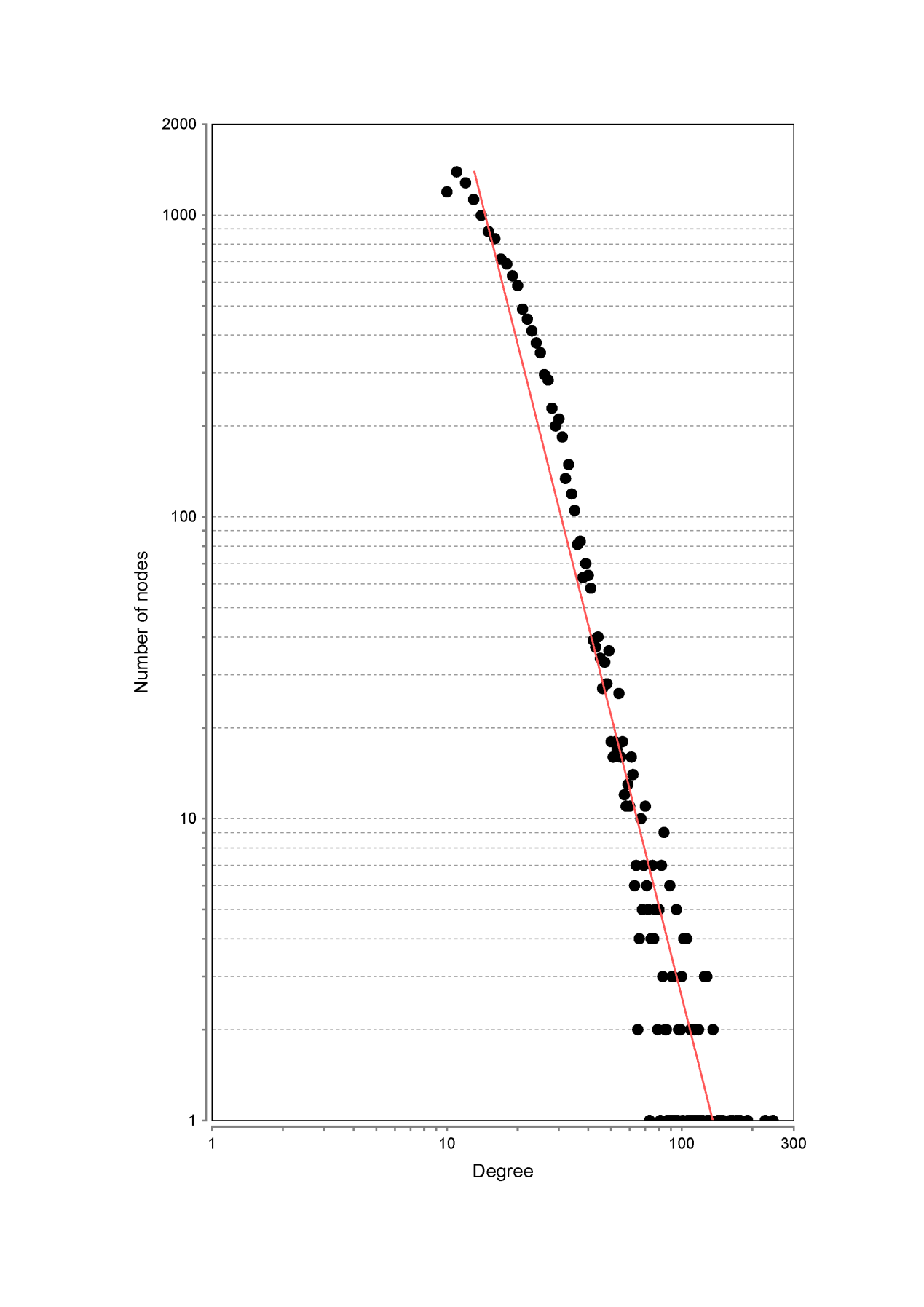


Figure S1. Power-law fit of the degrees and the number of nodes in the co-expression network of ovarian cancer.


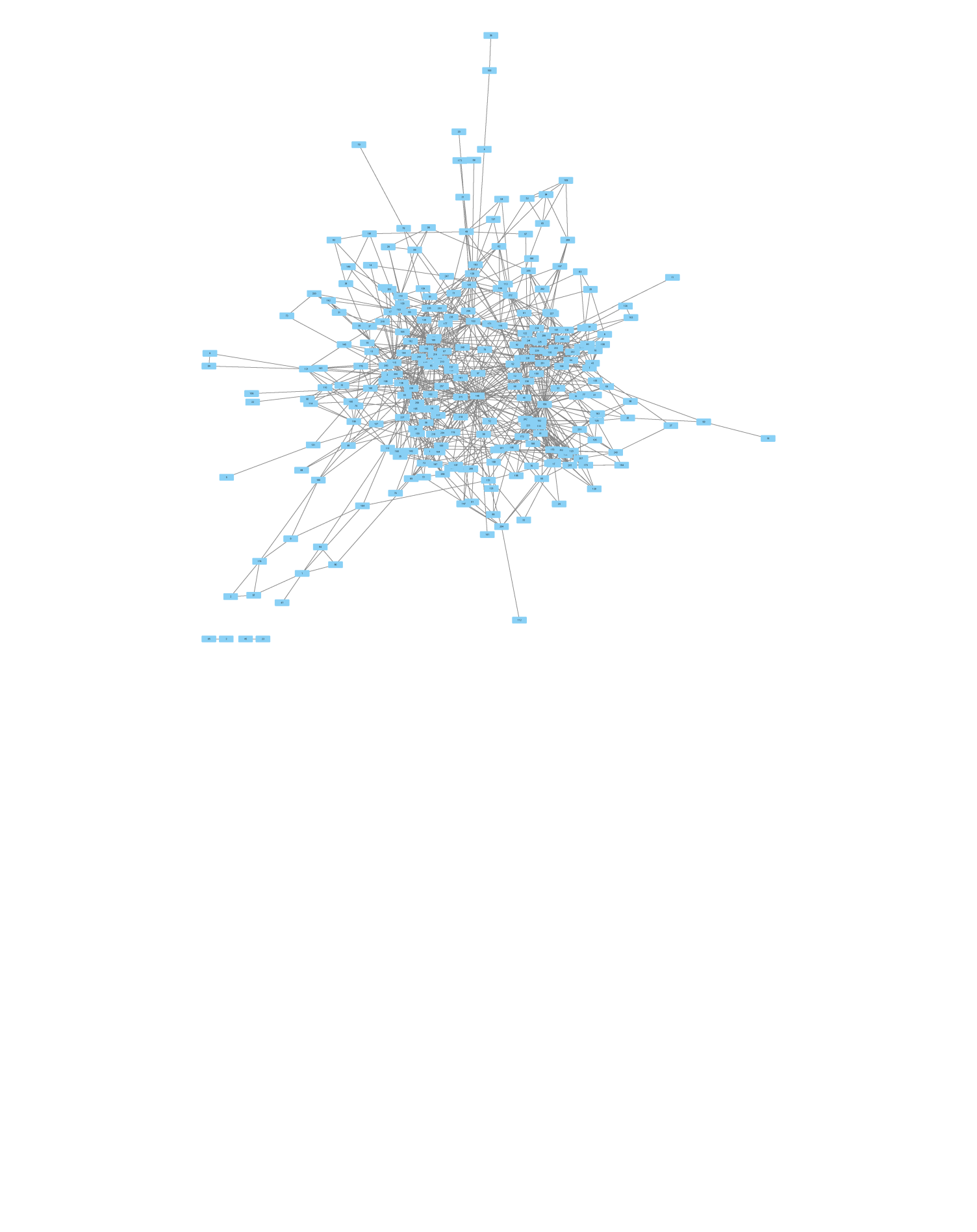


Figure S2. The module network of ovarian cancer.


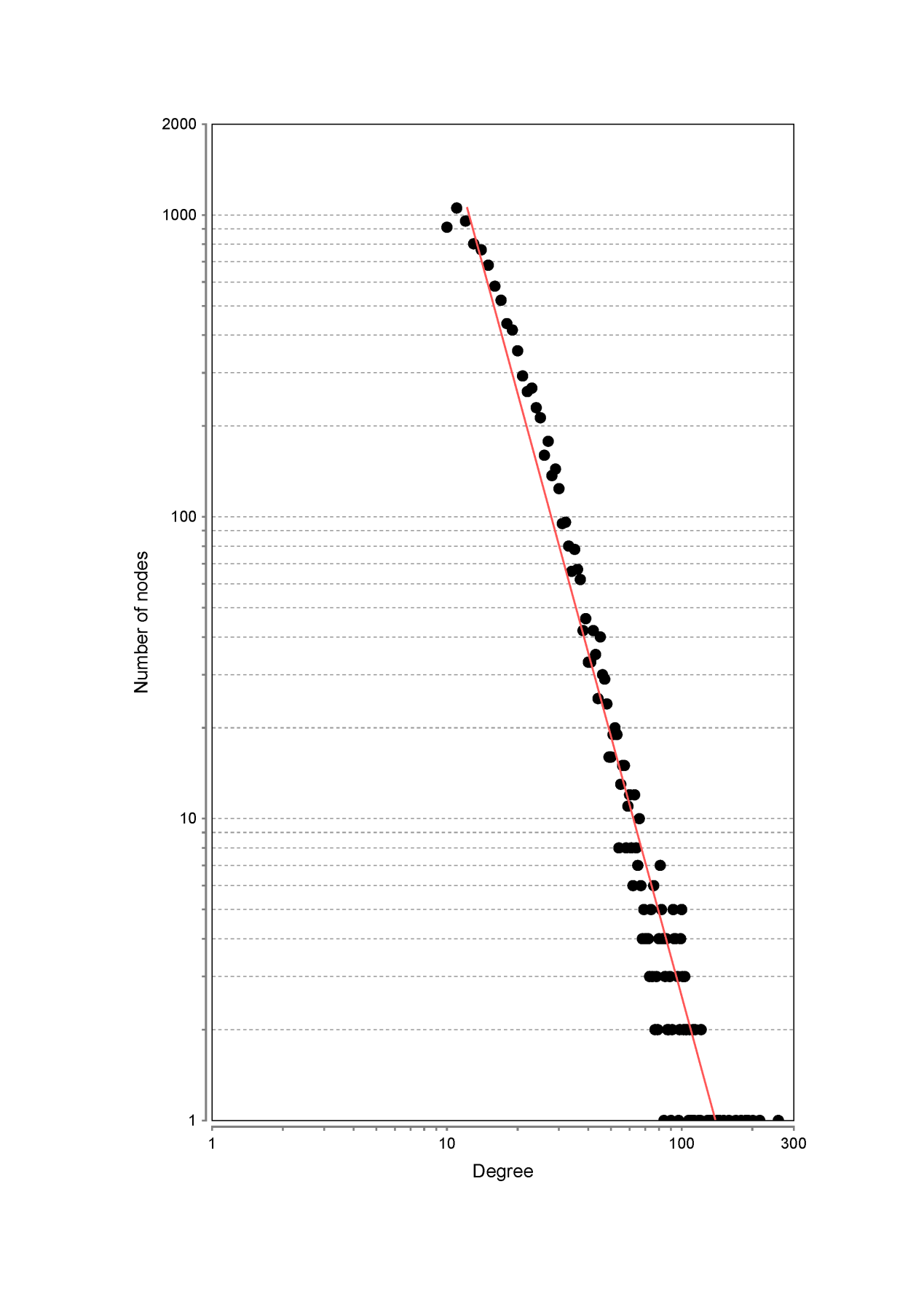


Figure S3. Power-law fit of the degrees and the number of nodes in the co-expression network of breast cancer.


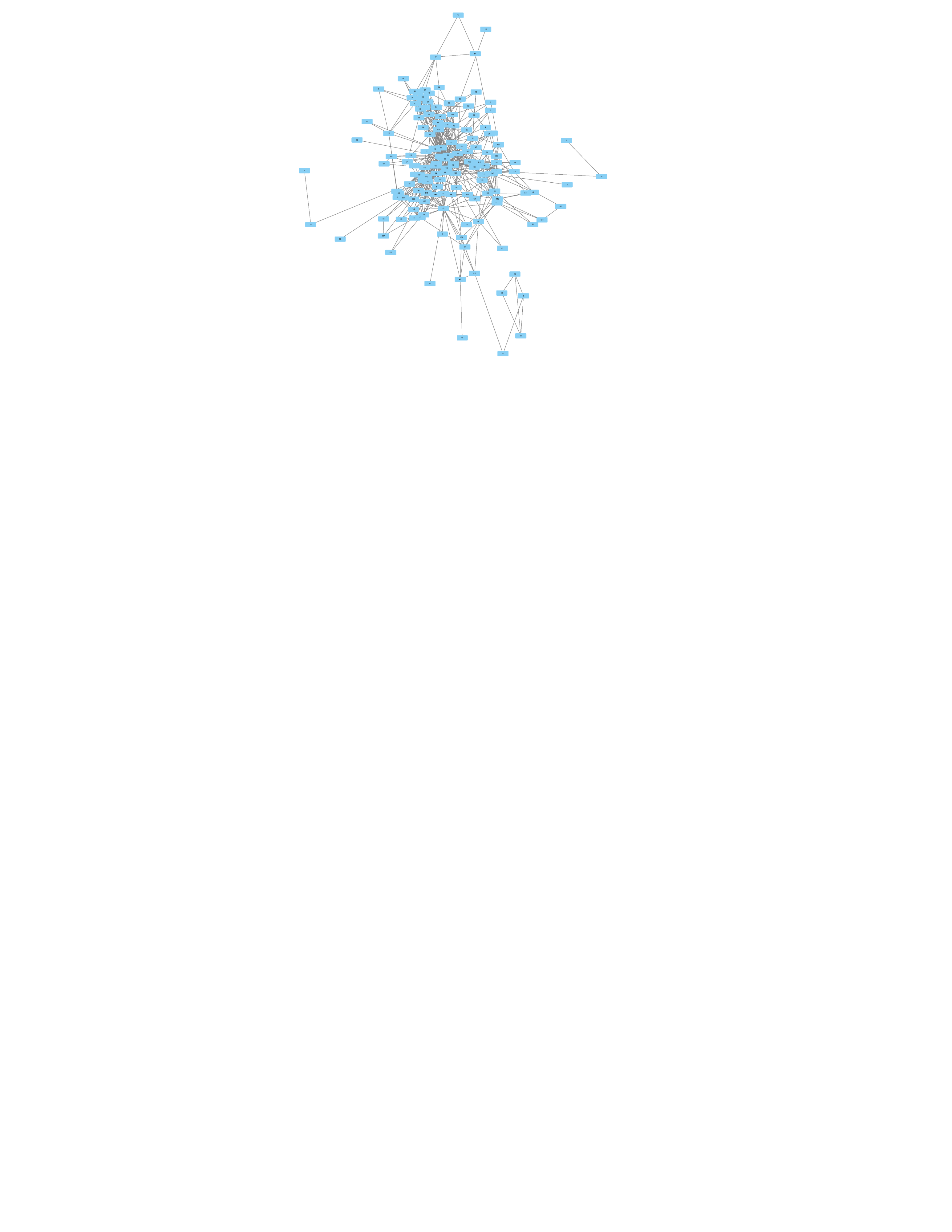


Figure S4. The module network of breast cancer.


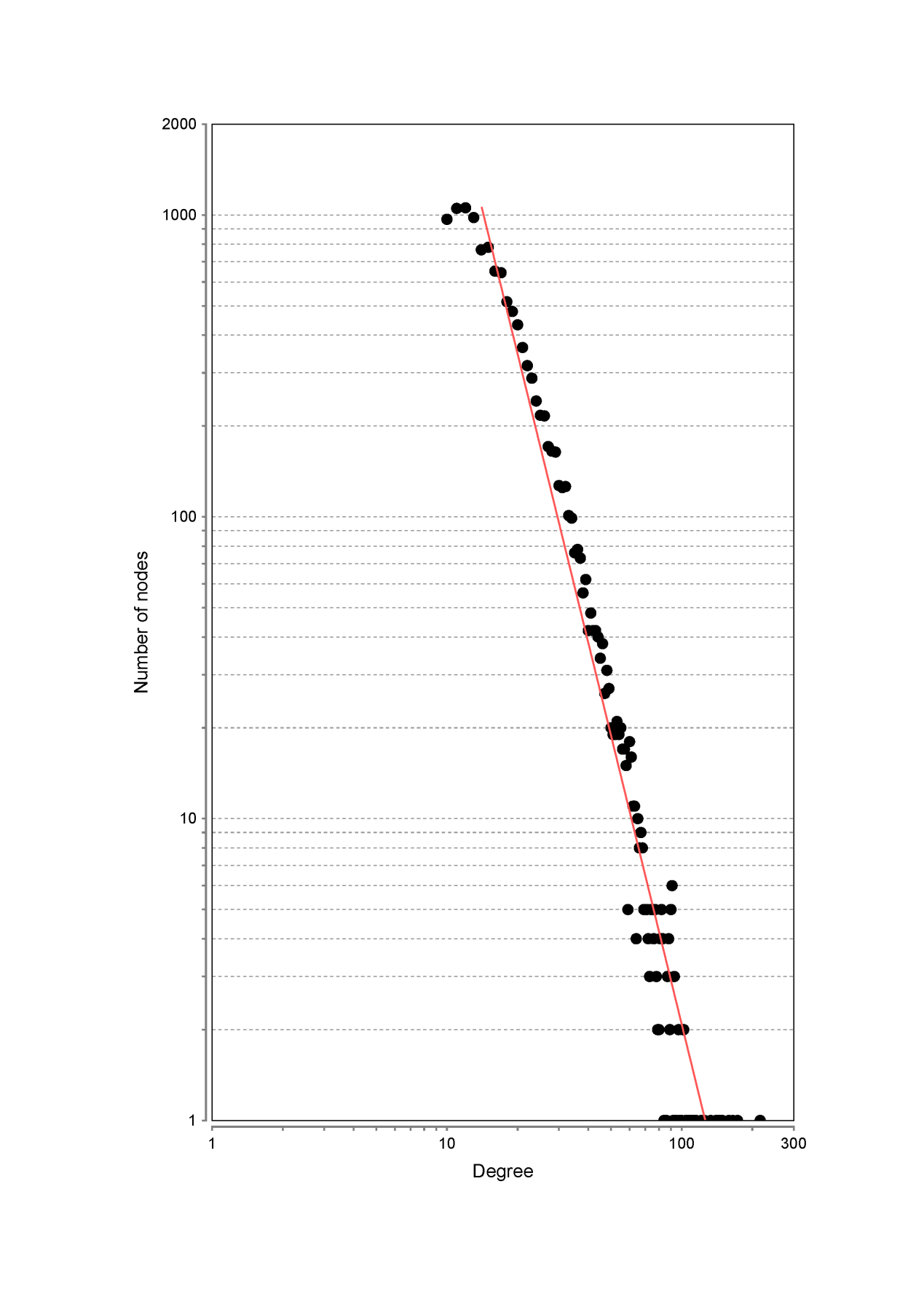


Figure S5. Power-law fit of the degrees and the number of nodes in the co-expression network of lung adenocarcinoma.


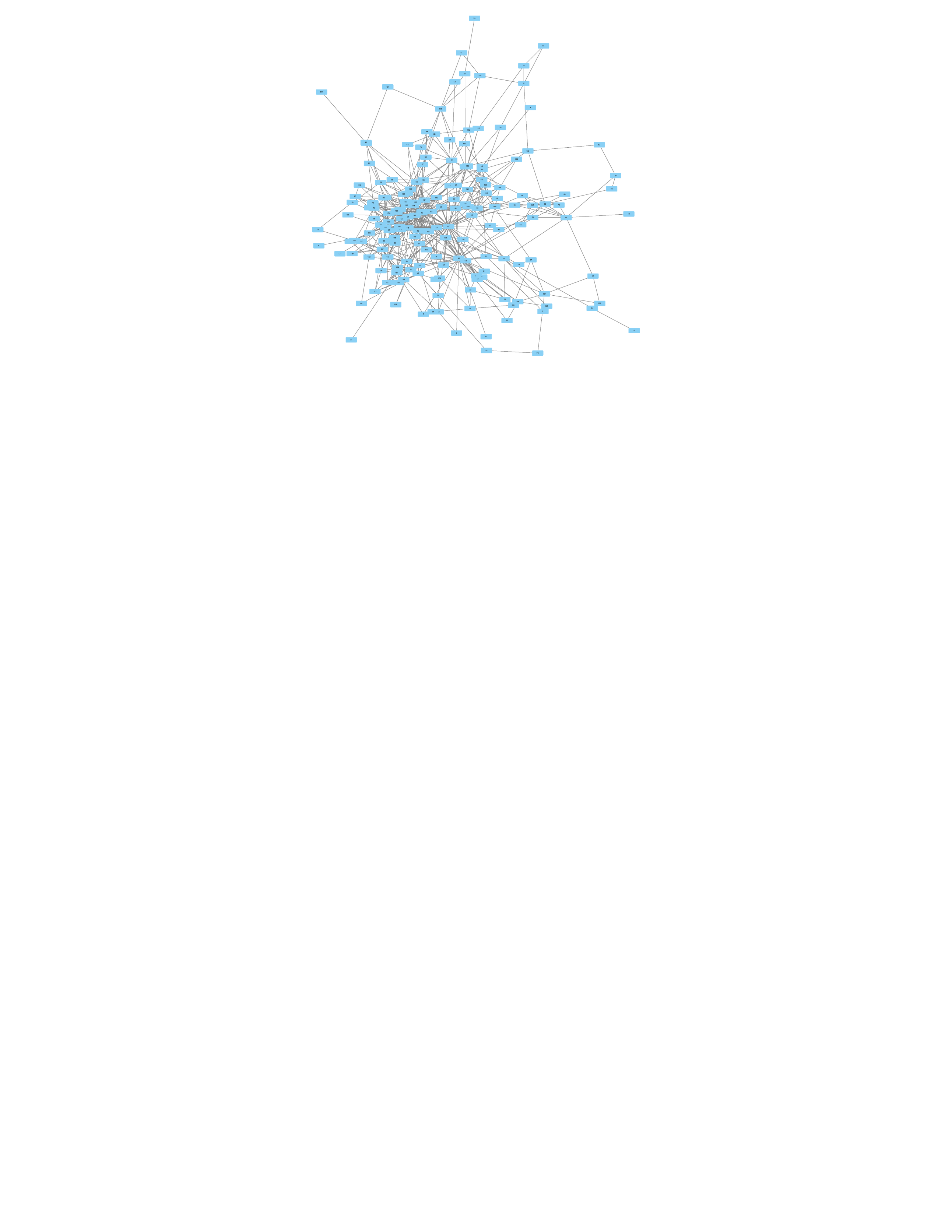


Figure S6. The module network of lung adenocarcinoma.

## Tables

| Table S1. Details of the ovarian cancer data sets | | | |
| --- | --- | --- | --- |
| Data set | Number of samples | Usage | Site |
| TCGA | 300 | Training | https://portal.gdc.cancer.gov/ |
| TCGA | 267 | Test | https://portal.gdc.cancer.gov/ |
| Merged set | 865 | Independent Test | http://kmplot.com/analysis/index.php?p=download |
| It should be mentioned that the TCGA data set was downloaded from http://tcga-data.nci.nih.gov/tcga/. And the source now is on <https://portal.gdc.cancer.gov/>. In the original version of the merged data set, there are 1287 samples. We removed the redundant samples which were collected from the TCGA, and there were 865 samples left. | | | |

| Table S2. The modules selected by GeneRank in ovarian cancer | |
| --- | --- |
| Module Id | Importance |
| 146 | 0.372335778 |
| 198 | 0.331120904 |
| 76 | 0.321422536 |
| 26 | 0.320376025 |
| 160 | 0.30058806 |
| 158 | 0.293835521 |
| 241 | 0.281424543 |
| 133 | 0.247145276 |
| 228 | 0.240394511 |
| 164 | 0.237773251 |
| 110 | 0.235239625 |
| 40 | 0.233511166 |
| 130 | 0.223129246 |
| 41 | 0.216593096 |
| 167 | 0.188912779 |
| 197 | 0.186558836 |
| 246 | 0.183526754 |
| 103 | 0.173202477 |
| 132 | 0.172898281 |
| 115 | 0.169588951 |
| 78 | 0.163912595 |
| 236 | 0.162946818 |
| 68 | 0.158842907 |
| 77 | 0.140283446 |
| 169 | 0.139452949 |
| 121 | 0.134330834 |
| 229 | 0.133860331 |
| 44 | 0.132865718 |
| 170 | 0.128786755 |
| 70 | 0.126525574 |
| 102 | 0.120882067 |
| 206 | 0.120657051 |
| 59 | 0.11793352 |
| 251 | 0.114591974 |
| 237 | 0.107849792 |
| 233 | 0.105697385 |
| 99 | 0.10069684 |
| 126 | 0.098106006 |
| 213 | 0.096603884 |
| 27 | 0.095475985 |
| 230 | 0.094308562 |
| 215 | 0.09394871 |
| 7 | 0.089837609 |
| 5 | 0.086340925 |
| 98 | 0.081774919 |
| 100 | 0.081585264 |
| 180 | 0.08072044 |
| 240 | 0.076387012 |
| 232 | 0.07445145 |
| 166 | 0.074247692 |
| 131 | 0.07184942 |
| 234 | 0.071123735 |
| 219 | 0.070174547 |
| 212 | 0.068988265 |
| 195 | 0.068214514 |
| 91 | 0.067378204 |
| 238 | 0.064694556 |
| 11 | 0.063923374 |
| 33 | 0.061308828 |
| 252 | 0.061015191 |
| 159 | 0.06040612 |
| 120 | 0.058389128 |
| 250 | 0.057576694 |
| 34 | 0.056826374 |
| 114 | 0.055827777 |
| 225 | 0.055275822 |
| 107 | 0.054458835 |
| 95 | 0.053156341 |
| 243 | 0.052671075 |
| 162 | 0.051417212 |
| 140 | 0.05022032 |
| 222 | 0.048673894 |
| 172 | 0.047807441 |
| 214 | 0.04744615 |
| 92 | 0.04580371 |
| 254 | 0.045766624 |
| 60 | 0.045452451 |
| 122 | 0.04350393 |
| 152 | 0.043431665 |
| 156 | 0.043151121 |
| 52 | 0.042727443 |
| 224 | 0.042456025 |
| 242 | 0.042253432 |
| 231 | 0.041806197 |
| 239 | 0.041704807 |
| 42 | 0.041650582 |
| 211 | 0.04075699 |
| 207 | 0.040426028 |
| 101 | 0.039412494 |
| 168 | 0.038988063 |
| 147 | 0.03875624 |
| 163 | 0.038711762 |
| 175 | 0.037225884 |
| 249 | 0.037145619 |
| 148 | 0.036908216 |
| 6 | 0.034480602 |
| 205 | 0.03416231 |
| 157 | 0.034157744 |
| 136 | 0.033882207 |
| 24 | 0.033435704 |
| 4 | 0.033418928 |
| 151 | 0.03303401 |
| 48 | 0.032775426 |
| 150 | 0.03236984 |
| 86 | 0.031646093 |
| 255 | 0.031433454 |
| 65 | 0.030937234 |
| 57 | 0.030811423 |
| 223 | 0.030592946 |
| 210 | 0.030548575 |
| 161 | 0.030544131 |
| 183 | 0.030192014 |
| 248 | 0.029829229 |
| 105 | 0.029769808 |
| 179 | 0.029734965 |
| 36 | 0.029302972 |
| 201 | 0.029246433 |
| 182 | 0.028657859 |
| 112 | 0.028516812 |
| 43 | 0.028352452 |
| 56 | 0.028313293 |
| 127 | 0.02703491 |
| 217 | 0.02656053 |
| 123 | 0.026307941 |
| 155 | 0.026237509 |
| 49 | 0.025835454 |
| 258 | 0.025827127 |
| 50 | 0.025709771 |
| 221 | 0.025669894 |
| 208 | 0.025493997 |
| 125 | 0.025393441 |
| 227 | 0.02526235 |
| 247 | 0.025157579 |
| 18 | 0.024992189 |
| 119 | 0.024542214 |
| 118 | 0.024221686 |
| 15 | 0.024188645 |
| 185 | 0.023732847 |
| 139 | 0.023035998 |
| 257 | 0.022488325 |
| 135 | 0.022102693 |
| 94 | 0.022021439 |
| 226 | 0.021751088 |
| 81 | 0.020998112 |
| 188 | 0.020934795 |
| 199 | 0.020453171 |
| 171 | 0.020339818 |
| 55 | 0.019876365 |
| 1 | 0.019102178 |
| 218 | 0.01905315 |
| 143 | 0.018851641 |
| 181 | 0.018749259 |
| 184 | 0.018749259 |
| 187 | 0.018369245 |
| 97 | 0.018330647 |
| 117 | 0.018220017 |
| 79 | 0.018151411 |
| 69 | 0.017951108 |
| 116 | 0.01758218 |
| 177 | 0.017461974 |
| 72 | 0.017393922 |
| 54 | 0.017348513 |
| 46 | 0.017317623 |
| 209 | 0.016887898 |
| 145 | 0.016811909 |
| 244 | 0.016789995 |
| 35 | 0.016788613 |
| 10 | 0.016615295 |
| 204 | 0.016539901 |
| 256 | 0.016507998 |
| 17 | 0.016301585 |
| 193 | 0.015692439 |
| 253 | 0.015662544 |
| 9 | 0.015617366 |
| 84 | 0.015617366 |
| 93 | 0.015592096 |
| 128 | 0.015317717 |
| 129 | 0.01530743 |
| 216 | 0.015144834 |
| 178 | 0.014588824 |
| 200 | 0.01429617 |
| 111 | 0.014190272 |
| 61 | 0.014134775 |
| 190 | 0.014011318 |
| 235 | 0.013829221 |
| 194 | 0.013769568 |
| 189 | 0.013767705 |
| 104 | 0.013490792 |
| 245 | 0.01346394 |
| 154 | 0.013376805 |
| 137 | 0.013355526 |
| 196 | 0.013221695 |
| 176 | 0.013219186 |
| 138 | 0.013208466 |
| 192 | 0.013139174 |
| 203 | 0.013063507 |
| 25 | 0.012997027 |
| 16 | 0.01298973 |
| 153 | 0.012410671 |
| 202 | 0.012370042 |
| 39 | 0.011861087 |
| 144 | 0.011804701 |
| 124 | 0.01180408 |
| 75 | 0.011531009 |
| 134 | 0.011236888 |
| 12 | 0.01079022 |
| 83 | 0.010089526 |
| 87 | 0.010088764 |
| 51 | 0.010038775 |
| 30 | 0.009892427 |
| 113 | 0.009834858 |
| 21 | 0.009439835 |
| 38 | 0.009041168 |
| 220 | 0.008730268 |
| 64 | 0.008624776 |
| 37 | 0.008580237 |
| 80 | 0.008530229 |
| 63 | 0.008504365 |
| 165 | 0.008397082 |
| 45 | 0.008361449 |
| 96 | 0.008183619 |
| 142 | 0.008143483 |
| 14 | 0.008046127 |
| 88 | 0.008036401 |
| 108 | 0.008032923 |
| 90 | 0.008029389 |
| 71 | 0.007864748 |
| 13 | 0.007705446 |
| 186 | 0.007624948 |
| 47 | 0.0075944 |
| 19 | 0.007302896 |
| 109 | 0.007199282 |
| 191 | 0.00718182 |
| 174 | 0.007127546 |
| 8 | 0.007066053 |
| 29 | 0.007011377 |
| 89 | 0.006864834 |
| 23 | 0.006601144 |
| 62 | 0.006600083 |
| 28 | 0.006594143 |
| 67 | 0.006573581 |
| 53 | 0.006364062 |
| 31 | 0.006360345 |
| 66 | 0.005874641 |
| 141 | 0.005760342 |
| 82 | 0.005212876 |
| 32 | 0.004722787 |
| 74 | 0.004661193 |
| 58 | 0.004070871 |
| 149 | 0.003960664 |
| 73 | 0.003649367 |
| 2 | 0.003529286 |
| 173 | 0.003516701 |
| 20 | 0.002757554 |
| 3 | 0.002493766 |
| 85 | 0.002493766 |
| 22 | 0.002258722 |
| 106 | 0.002258722 |
| The top 13 modules were selected by our method | |

| Table S3. The modules selected by GeneRank in breast cancer | |
| --- | --- |
| Module Id | Importance |
| 75 | 0.709615948 |
| 10 | 0.565298184 |
| 68 | 0.524714986 |
| 37 | 0.51835736 |
| 132 | 0.46781975 |
| 18 | 0.454975024 |
| 120 | 0.438042881 |
| 95 | 0.436738283 |
| 33 | 0.416112122 |
| 84 | 0.399277303 |
| 62 | 0.350369348 |
| 85 | 0.328599153 |
| 13 | 0.327288274 |
| 31 | 0.309961091 |
| 96 | 0.30880227 |
| 27 | 0.308761854 |
| 115 | 0.306271064 |
| 52 | 0.279217128 |
| 117 | 0.277037531 |
| 114 | 0.25487074 |
| 69 | 0.24388774 |
| 58 | 0.238562402 |
| 41 | 0.225015665 |
| 137 | 0.199518874 |
| 92 | 0.197237153 |
| 139 | 0.196340508 |
| 56 | 0.183164189 |
| 76 | 0.176187482 |
| 124 | 0.175993507 |
| 110 | 0.174290626 |
| 134 | 0.154753843 |
| 127 | 0.152767455 |
| 145 | 0.143739925 |
| 77 | 0.138934315 |
| 104 | 0.136548818 |
| 148 | 0.128410844 |
| 94 | 0.12566669 |
| 140 | 0.120976833 |
| 46 | 0.119221356 |
| 130 | 0.11225548 |
| 138 | 0.108692093 |
| 61 | 0.107409686 |
| 131 | 0.105094976 |
| 143 | 0.103892351 |
| 91 | 0.100859563 |
| 112 | 0.097197187 |
| 106 | 0.096780107 |
| 28 | 0.09595868 |
| 51 | 0.095515937 |
| 34 | 0.092471222 |
| 89 | 0.091460992 |
| 64 | 0.090189203 |
| 88 | 0.086425679 |
| 126 | 0.085993531 |
| 93 | 0.083182717 |
| 90 | 0.082596773 |
| 142 | 0.079303818 |
| 133 | 0.077438009 |
| 118 | 0.076144728 |
| 49 | 0.074830616 |
| 78 | 0.070596682 |
| 7 | 0.069495762 |
| 144 | 0.063693209 |
| 98 | 0.063418927 |
| 39 | 0.0621578 |
| 121 | 0.060924718 |
| 111 | 0.05945883 |
| 59 | 0.058942947 |
| 107 | 0.058798817 |
| 50 | 0.058210114 |
| 43 | 0.058210114 |
| 108 | 0.058042186 |
| 149 | 0.057961329 |
| 74 | 0.056637286 |
| 35 | 0.054933727 |
| 101 | 0.053465874 |
| 122 | 0.053088943 |
| 57 | 0.052870431 |
| 119 | 0.050978109 |
| 128 | 0.050566063 |
| 103 | 0.049946865 |
| 116 | 0.049610381 |
| 48 | 0.04946751 |
| 36 | 0.047458786 |
| 20 | 0.046361458 |
| 97 | 0.046345972 |
| 67 | 0.045507511 |
| 21 | 0.045323588 |
| 24 | 0.044793906 |
| 72 | 0.044477883 |
| 73 | 0.044325678 |
| 86 | 0.044184864 |
| 135 | 0.04291347 |
| 55 | 0.042730696 |
| 113 | 0.042458302 |
| 8 | 0.042204249 |
| 15 | 0.040542376 |
| 66 | 0.038157327 |
| 146 | 0.037729179 |
| 11 | 0.036785569 |
| 150 | 0.036708589 |
| 38 | 0.035709002 |
| 45 | 0.034526966 |
| 87 | 0.034358984 |
| 123 | 0.034032837 |
| 70 | 0.032034107 |
| 1 | 0.03140625 |
| 109 | 0.030673317 |
| 65 | 0.030478697 |
| 129 | 0.030417681 |
| 47 | 0.029797003 |
| 53 | 0.028624416 |
| 71 | 0.027746464 |
| 63 | 0.027530044 |
| 100 | 0.026398481 |
| 147 | 0.026271031 |
| 105 | 0.024329833 |
| 40 | 0.024028591 |
| 136 | 0.023865992 |
| 9 | 0.023845552 |
| 99 | 0.023657618 |
| 29 | 0.023505037 |
| 83 | 0.020172313 |
| 16 | 0.018755276 |
| 17 | 0.018623973 |
| 26 | 0.016620879 |
| 30 | 0.016033198 |
| 42 | 0.01493522 |
| 23 | 0.014484889 |
| 12 | 0.014250117 |
| 3 | 0.01390549 |
| 79 | 0.013011644 |
| 2 | 0.012761127 |
| 54 | 0.012377336 |
| 125 | 0.011292349 |
| 141 | 0.010322648 |
| 81 | 0.009762573 |
| 102 | 0.008799396 |
| 32 | 0.008412459 |
| 82 | 0.00832872 |
| 4 | 0.007728558 |
| 25 | 0.006916347 |
| 14 | 0.005808972 |
| 5 | 0.005735671 |
| 44 | 0.005608392 |
| 60 | 0.004881884 |
| 6 | 0.00395107 |
| 19 | 0.00320203 |
| 22 | 0.00320203 |
| 80 | 0.00224241 |
| The top 8 modules were selected by our method | |

| Table S4. The modules selected by GeneRank in lung adenocarcinoma | | | | | |  |  |  |
| --- | --- | --- | --- | --- | --- | --- | --- | --- |
| Module Id | | Importance | | | |  |  |  |
| 147 | | 0.631702028 | | | |  |  |  |
| 67 | | 0.618525145 | | | |  |  |  |
| 146 | | 0.475867129 | | | |  |  |  |
| 154 | | 0.42897414 | | | |  |  |  |
| 155 | | 0.415235583 | | | |  |  |  |
| 35 | | 0.409175566 | | | |  |  |  |
| 92 | | 0.38900173 | | | |  |  |  |
| 152 | | 0.388527261 | | | |  |  |  |
| 47 | | 0.375196141 | | | |  |  |  |
| 141 | | 0.353309804 | | | |  |  |  |
| 48 | | 0.352132128 | | | |  |  |  |
| 108 | | 0.338622818 | | | |  |  |  |
| 76 | | 0.334792024 | | | |  |  |  |
| 113 | | 0.324107365 | | | |  |  |  |
| 126 | | 0.313073817 | | | |  |  |  |
| 21 | | 0.306488236 | | | |  |  |  |
| 45 | | 0.303623241 | | | |  |  |  |
| 158 | | 0.292924043 | | | |  |  |  |
| 38 | | 0.290651106 | | | |  |  |  |
| 75 | | 0.28243779 | | | |  |  |  |
| 49 | | 0.279825118 | | | |  |  |  |
| 174 | | 0.278391444 | | | |  |  |  |
| 109 | | 0.269035467 | | | |  |  |  |
| 124 | | 0.263322682 | | | |  |  |  |
| 160 | | 0.244271326 | | | |  |  |  |
| 88 | | 0.243332676 | | | |  |  |  |
| 9 | | 0.236434313 | | | |  |  |  |
| 81 | | 0.229359244 | | | |  |  |  |
| 166 | | 0.221330274 | | | |  |  |  |
| 181 | | 0.207318559 | | | |  |  |  |
| 40 | | 0.205252783 | | | |  |  |  |
| 82 | | 0.200519721 | | | |  |  |  |
| 167 | | 0.1990234 | | | |  |  |  |
| 17 | | 0.196502128 | | | |  |  |  |
| 135 | | 0.193921402 | | | |  |  |  |
| 137 | | 0.181318088 | | | |  |  |  |
| 103 | | 0.174111068 | | | |  |  |  |
| 150 | | 0.168877376 | | | |  |  |  |
| 32 | | 0.165866797 | | | |  |  |  |
| 121 | | 0.156259762 | | | |  |  |  |
| 104 | | 0.153451278 | | | |  |  |  |
| 170 | | 0.150401089 | | | |  |  |  |
| 180 | | 0.1494835 | | | |  |  |  |
| 128 | | 0.147969241 | | | |  |  |  |
| 168 | | 0.144361153 | | | |  |  |  |
| 22 | | 0.140668424 | | | |  |  |  |
| 54 | | 0.131897379 | | | |  |  |  |
| 41 | | 0.128688284 | | | |  |  |  |
| 123 | | 0.128658715 | | | |  |  |  |
| 57 | | 0.12740167 | | | |  |  |  |
| 165 | | 0.121088839 | | | |  |  |  |
| 87 | | 0.119858821 | | | |  |  |  |
| 59 | | 0.118289513 | | | |  |  |  |
| 157 | | 0.113673629 | | | |  |  |  |
| 98 | | 0.11309593 | | | |  |  |  |
| 153 | | 0.112520326 | | | |  |  |  |
| 169 | | 0.111098731 | | | |  |  |  |
| 143 | | 0.106957174 | | | |  |  |  |
| 163 | | 0.106828714 | | | |  |  |  |
| 5 | | 0.105264832 | | | |  |  |  |
| 72 | | 0.105001469 | | | |  |  |  |
| 127 | | 0.10429063 | | | |  |  |  |
| 60 | | 0.100813011 | | | |  |  |  |
| 161 | | 0.099692301 | | | |  |  |  |
| 114 | | 0.096597706 | | | |  |  |  |
| 44 | | 0.095656457 | | | |  |  |  |
| 175 | | 0.095328257 | | | |  |  |  |
| 172 | | 0.094553929 | | | |  |  |  |
| 148 | | 0.092312239 | | | |  |  |  |
| 83 | | 0.090521455 | | | |  |  |  |
| 151 | | 0.090245678 | | | |  |  |  |
| 70 | | 0.088808981 | | | |  |  |  |
| 162 | | 0.087356489 | | | |  |  |  |
| 73 | | 0.083954377 | | | |  |  |  |
| 164 | | 0.082331099 | | | |  |  |  |
| 93 | | 0.079590582 | | | |  |  |  |
| 24 | | 0.078633609 | | | |  |  |  |
| 173 | | 0.076414516 | | | |  |  |  |
| 107 | | 0.073134722 | | | |  |  |  |
| 129 | | 0.070264207 | | | |  |  |  |
| 79 | | 0.069701314 | | | |  |  |  |
| 144 | | 0.06959838 | | | |  |  |  |
| 149 | | 0.067233 | | | |  |  |  |
| 122 | | 0.066717194 | | | |  |  |  |
| 101 | | 0.065434677 | | | |  |  |  |
| 105 | | 0.063719277 | | | |  |  |  |
| 116 | | 0.063569314 | | | |  |  |  |
| 117 | | 0.0630363 | | | |  |  |  |
| 179 | | 0.062776964 | | | |  |  |  |
| 29 | | 0.062237286 | | | |  |  |  |
| 171 | | 0.062152288 | | | |  |  |  |
| 125 | | 0.056601319 | | | |  |  |  |
| 94 | | 0.05659213 | | | |  |  |  |
| 115 | | 0.055238527 | | | |  |  |  |
| 145 | | 0.054579872 | | | |  |  |  |
| 78 | | 0.054004634 | | | |  |  |  |
| 46 | | 0.052191196 | | | |  |  |  |
| 14 | | 0.051896338 | | | |  |  |  |
| 84 | | 0.051033593 | | | |  |  |  |
| 85 | | 0.050919751 | | | |  |  |  |
| 89 | | 0.049749372 | | | |  |  |  |
| 119 | | 0.048943208 | | | |  |  |  |
| 95 | | 0.048196726 | | | |  |  |  |
| 133 | | 0.04787996 | | | |  |  |  |
| 136 | | 0.047419207 | | | |  |  |  |
| 25 | | 0.047297882 | | | |  |  |  |
| 15 | | 0.046247072 | | | |  |  |  |
| 132 | | 0.046034069 | | | |  |  |  |
| 159 | | 0.045961741 | | | |  |  |  |
| 56 | | 0.045491449 | | | |  |  |  |
| 90 | | 0.045386105 | | | |  |  |  |
| 106 | | 0.045334484 | | | |  |  |  |
| 177 | | 0.044367521 | | | |  |  |  |
| 31 | | 0.044099662 | | | |  |  |  |
| 69 | | 0.043278377 | | | |  |  |  |
| 50 | | 0.041933884 | | | |  |  |  |
| 64 | | 0.039977186 | | | |  |  |  |
| 51 | | 0.039621338 | | | |  |  |  |
| 120 | | 0.039408639 | | | |  |  |  |
| 77 | | 0.039263621 | | | |  |  |  |
| 55 | | 0.03837031 | | | |  |  |  |
| 28 | | 0.038299364 | | | |  |  |  |
| 156 | | 0.037653121 | | | |  |  |  |
| 91 | | 0.03695286 | | | |  |  |  |
| 86 | | 0.036345997 | | | |  |  |  |
| 20 | | 0.034028345 | | | |  |  |  |
| 178 | | 0.033638943 | | | |  |  |  |
| 12 | | 0.033290494 | | | |  |  |  |
| 52 | | 0.033088205 | | | |  |  |  |
| 39 | | 0.032953188 | | | |  |  |  |
| 112 | | 0.032672567 | | | |  |  |  |
| 33 | | 0.03173932 | | | |  |  |  |
| 97 | | 0.031607823 | | | |  |  |  |
| 23 | | 0.030475186 | | | |  |  |  |
| 16 | | 0.02943259 | | | |  |  |  |
| 96 | | 0.029179194 | | | |  |  |  |
| 142 | | 0.029119875 | | | |  |  |  |
| 74 | | 0.028301744 | | | |  |  |  |
| 110 | | 0.028027987 | | | |  |  |  |
| 118 | | 0.027516284 | | | |  |  |  |
| 130 | | 0.027419521 | | | |  |  |  |
| 27 | | 0.026600206 | | | |  |  |  |
| 36 | | 0.025572544 | | | |  |  |  |
| 176 | | 0.024497905 | | | |  |  |  |
| 10 | | 0.02392135 | | | |  |  |  |
| 80 | | 0.023853011 | | | |  |  |  |
| 37 | | 0.0237225 | | | |  |  |  |
| 62 | | 0.023477722 | | | |  |  |  |
| 61 | | 0.023252988 | | | |  |  |  |
| 134 | | 0.022708097 | | | |  |  |  |
| 66 | | 0.022698479 | | | |  |  |  |
| 140 | | 0.022553222 | | | |  |  |  |
| 139 | | 0.022329857 | | | |  |  |  |
| 34 | | 0.021588828 | | | |  |  |  |
| 19 | | 0.021401041 | | | |  |  |  |
| 102 | | 0.02134897 | | | |  |  |  |
| 26 | | 0.021118822 | | | |  |  |  |
| 100 | | 0.020809001 | | | |  |  |  |
| 68 | | 0.020665308 | | | |  |  |  |
| 63 | | 0.020633592 | | | |  |  |  |
| 53 | | 0.018028484 | | | |  |  |  |
| 131 | | 0.016712443 | | | |  |  |  |
| 7 | | 0.016087904 | | | |  |  |  |
| 43 | | 0.015742648 | | | |  |  |  |
| 99 | | 0.014744598 | | | |  |  |  |
| 6 | | 0.014615598 | | | |  |  |  |
| 58 | | 0.01422318 | | | |  |  |  |
| 65 | | 0.013805574 | | | |  |  |  |
| 2 | | 0.013678265 | | | |  |  |  |
| 71 | | 0.011620713 | | | |  |  |  |
| 111 | | 0.01016075 | | | |  |  |  |
| 138 | | 0.009699757 | | | |  |  |  |
| 4 | | 0.008054897 | | | |  |  |  |
| 42 | | 0.007595143 | | | |  |  |  |
| 13 | | 0.007444082 | | | |  |  |  |
| 8 | | 0.006922082 | | | |  |  |  |
| 11 | | 0.005402157 | | | |  |  |  |
| 18 | | 0.002244389 | | | |  |  |  |
| 1 | | 0.00074813 | | | |  |  |  |
| 3 | | 0.00074813 | | | |  |  |  |
| 30 | | 0.00074813 | | | |  |  |  |
| The top 9 modules were selected by our method. | | | | | |  |  |  |
| Table S5. Survival analysis of the control modules in ovarian cancer data sets | | | | | | | | |
|  | HR | | HR- | HR+ | Logrank p-value | |  | Samples in bad-outcome group |
| Test data set | 1.56 | | 1.12 | 2.19 | 0.0054 | | 133 | 134 |
| Independent data set | 1.53 | | 1.27 | 1.84 | 3.06E-06 | |  |  |

| Table S6. Survival analysis of the published signature in ovarian cancer data sets | | | | | |
| --- | --- | --- | --- | --- | --- |
|  | HR | HR- | HR+ | Logrank p-value |  |
| Test data set | 1.53 | 1.10 | 2.12 | 0.0076 |  |
| Independent data set | 1.19 | 0.99 | 1.42 | 0.037 |  |

| Table S7. Survival analysis of the 42-gene signature in ovarian cancer data sets | | | | | |
| --- | --- | --- | --- | --- | --- |
|  | HR | HR- | HR+ | Logrank p-value |  |
| Test data set | 1.44 | 1.03 | 2.01 | 0.021 |  |
| Independent data set | 1.02 | 0.85 | 1.22 | 0.43 |  |

| Table S8. Survival analysis of the control modules in breast cancer data sets | | | | | | | |
| --- | --- | --- | --- | --- | --- | --- | --- |
|  | HR | HR- | HR+ | Logrank p-value |  |  |  |
| Test data set | 1.38 | 0.98 | 1.94 | 0.046 | 134 | | 135 |
| Independent data set | 2.14 | 1.41 | 3.26 | 0.00019 | 143 | | 143 |
|  | | | | |  |  |  |

| Table S9. Survival analysis of the published signature in breast cancer data sets | | | | | | |
| --- | --- | --- | --- | --- | --- | --- |
|  | HR | HR- | HR+ | Logrank p-value |  | Samples in bad-outcome group |
| Test data set | 1.58 | 1.12 | 2.23 | 0.0048 | 134 | 135 |
| Independent data set | 1.70 | 1.13 | 2.56 | 0.0066 | 143 | 143 |

| Table S10. Survival analysis of the 42-gene signature in breast cancer data sets | | | | | | |
| --- | --- | --- | --- | --- | --- | --- |
|  | HR | HR- | HR+ | Logrank p-value |  | Samples in bad-outcome group |
| Test data set | 1.2 | 0.86 | 1.70 | 0.15 | 117 | 118 |
| Independent data set | 0.73 | 0.55 | 0.98 | 0.018 | 221 | 222 |

|  |
| --- |

| Table S11. Survival analysis of the control modules in Lung adenocarcinoma data sets | | | | | |
| --- | --- | --- | --- | --- | --- |
|  | HR | HR- | HR+ | Logrank p-value |  |
| Test data set | 2.23 | 1.43 | 3.48 | 1.63E-04 |  |
| Independent data set | 1.19 | 0.92 | 1.55 | 0.11 |  |

| Table S12. Survival analysis of the published signature in Lung adenocarcinoma data sets | | | | | | |
| --- | --- | --- | --- | --- | --- | --- |
|  | HR | HR- | HR+ | Logrank p-value |  | Samples in bad-outcome group |
| Test data set | 1.14 | 0.75 | 1.75 | 0.35 | 117 | 118 |
| Independent data set | 0.94 | 0.72 | 1.21 | 0.39 | 221 | 222 |

| Table S13. Survival analysis of the 42-gene signature in Lung adenocarcinoma data sets | | | | | |
| --- | --- | --- | --- | --- | --- |
|  | HR | HR- | HR+ | Logrank p-value |  |
| Test data set | 1.72 | 1.12 | 2.65 | 0.0098 |  |
| Independent data set | 1.27 | 0.98 | 1.64 | 0.066 |  |
